# An extended approach to impact assessment in the Horizon 2020 digital manufacturing domain

**DOI:** 10.12688/openreseurope.16235.1

**Published:** 2024-01-08

**Authors:** Nicholas Fair, Stefano Modafferi, Briony Gray, Jun Chan, Francesco Lelli

**Affiliations:** 1 IT Innovation, University of Southampton, Southampton, England, UK; 2 School of Economics and Management, Tilburg University, Tilburg, North Brabant, The Netherlands

**Keywords:** impact assessment, Horizon2020, Industry4.0, outcome indicators, business value

## Abstract

This paper presents an extended approach to Impact Assessment (IA) within European Union funded large-scale projects within the manufacturing domain, which may offer value to other research projects and SME organisations seeking to develop detailed organizational reporting. It details the six-phase process that forms the framework for this extended approach, demonstrating how project Outcome Indictors and impact assessment criterion can be aligned through an extensive review and integration of existing impact domains, objectives, measures and evidence sources with project documentation to provide the detailed individual impact assessment criteria for this extended IA approach. It also reports on the application of the approach in the EC-funded digital manufacturing project, European Connected Factory Platform for Agile Manufacturing (EFPF), finding that 24 of the 27 IA criteria were met or exceed, suggesting that the project made an important contribution to the EU Industry4.0 ecosystem through furthering the key priorities of Industrial Leadership, Data Integration, Uptake of New Technologies, Open Science, the Circulation of Knowledge, and a minor contribution to Climate Change Mitigation.

## Plain language summary

This paper details an extended approach to Impact Assessment within Horizon Innovation projects. It extends the standard methods deployed within Horizon projects for impact assessment by presenting a phased methodology involving identifying and aligning project KPIs and data sources with established impact assessment domains, objectives, and measures, before collecting data at timely points through detailed surveys, and then analysing the results. The end result is an extensive list of specific, measurable impact assessment criteria linked to project KPIs and outcomes with attached data sources, making it easy to design impact assessment data collection surveys that return readily comparable results even when responses are collected several years apart.

## Introduction

The ‘Industry4.0’ concept; a subset of Klaus Schwab’s fourth industrial revolution ( Schwab, 2017), is characterised by the integration of smart technologies, machine-to-machine communication, internet of things, and artificial intelligence and machine learning into the manufacturing domain and production systems ( Gerrikagoitia *et al*., 2019). Advances in Computer Science are already supporting this transformation, mostly through recent developments in AI, resulting in a variety of approaches, data sources, methods, knowledge, and tools to support it ( Holmstrom & Partanen, 2014; Kovacova & Lazaroiu, 2021; Wajid & Bhullar, 2019). In addition, digital platforms are now playing an increasingly important role in integrating new technologies and services within the I4.0 domain ( Urbach & Roglinger, 2019), especially for SME manufacturers, by providing the underpinning systems and architectures that SMEs may lack. These allow for SME manufacturing organizations to rethink their business processes, systems, outputs and needs, and to digitize and sustain these in the modern day ( Kovacova & Lazaroiu, 2021). In recent years, this concept has evolved into ‘Industry5.0’ (I5.0), where a greater emphasis is placed on digital technologies being deployed to enhance human knowledge, skills, and actions than I4.0. I5.0 “is value-driven” ( Breque *et al.*, 2021), which means that it recognises wider goals for manufacturing than just the production of an item and economic growth. It values the knowledge and skills of the humans in the system, while also recognising that manufacturing processes and companies are embedded in the wider context of a society and therefore need to reflect and promote societal goals, such as prosperity, resilience, environmental sustainability, inclusivity, equality, and mental and physical health & wellbeing ( Xu *et al.*, 2021). In short, I5.0 conceptualises digital manufacturing as a sociotechnical system (e.g. Bijker, 1997; Cummings, 1978; Geels, 2002; Geels, 2004; Oudshoorn & Pinch, 2003; Trist, 1981) which recognises the ‘interlinkages’ ( Geels, 2004) between technologies, knowledge, networks of people, organisations, cultural norms and societal goals, capital, and standards, rules & regulations ( Fair & Modafferi, 2022) that lead to ‘co-evolution’ effects ( Geels, 2002).

As a sociotechnical system, digital manufacturing in the EU is driven by real-time and specific demand for new or enhanced products and services ( Moller, 2016) often in small lot sizes, as well as by the need for process optimisation, waste and defect reductions, employee well-being, and production efficiencies. Since the turn of the millennium, this has resulted in the development and uptake of new information technology (IT) systems in manufacturing processes with tangible benefits (ibid.). Firstly, time and manufacturing costs have been reduced, with a greater emphasis on the optimisation stage before production ( Chryssolouris *et al*., 2009). Secondly, data and the integration of knowledge throughout the manufacturing process have reduced errors and aided productivity ( Wajid & Bhullar, 2019). Thirdly, decentralised manufacturing has increased variety in the products and services available ( Holmstrom & Partanen, 2014). Finally, manufacturers and small and mid-sized enterprises (SMEs) have been better able to identify their core competencies, IT demands, and potential novel solutions that digital manufacturing methods may offer ( Gerrikagoitia *et al*., 2019).

Such IT demands for novel solutions has meant that digital platforms for manufacturing play a key role for SME manufacturers in addressing competitive pressures and integrating new technologies and services. However, challenges remain in the technological domain (e.g. making full use of new technologies that enable SMEs to meet the requirements of evolving supply and value chains ( Bosman *et al.*, 2019)); the social domain (e.g. optimising and utilising the relevant tools, skills and working methods with the human capital an SME has, or developing appropriate working guidelines for advanced IT deployments ( Urbach & Roglinger, 2019)); the industrial domain (e.g. sustainability, digital innovation and adaptation ( Savastano *et al*., 2018)); and, finally, in the environmental domain (e.g. new environmental objectives such as climate change mitigation and waste reduction (UN SDGs)).

## Impact assessment and related work in the digital manufacturing domain

As a result, assessing the impact of I4.0 (or I5.0) deployments in the relevant domains and with sufficient depth, including among others the provision and use of digital platforms, is significant, not only from the point of view of success, but also in relation to organisational research methods as they span the area between research development and organisational development. However, the concept and methods for conducting ‘impact assessment’ are not uniform, varying from domain to domain and objective to objective. For example, the International Federation of Red Cross/Red Crescent Societies (IFRC) define IA as “a means of measuring the effectiveness of organisational activities and judging the significance of changes brought about by those activities” ( IFRC, 2005), whereas the International Association for Impact Assessment ( IAIA, 2022) argues that IA is “a structured a process for considering the implications, for people and their environment, of proposed actions while there is still an opportunity to modify (or even, if appropriate, abandon) the proposals. It is applied at all levels of decision-making, from policies to specific projects.” The Organisation for Economic Co-operation and Development (OECD) recognises this divergence by suggesting that IA can be both ‘ex ante’ (a planning task based on predicted impact enabling modifications) and ‘ex post’ (an evaluation task focussing on the effects of an activity in terms of change) ( OECD, 2015). Furthermore, for IA in the manufacturing domain, as with other domains, impact itself is a problematic concept ( Gerrikagoitia *et al*., 2019) as it implies a “simple, linear relationship” ( OECD, 2015, p.3) between research outputs and social change, or in this case, between I4.0 developments in technologies, tools and services and manufacturing gains in productivity, efficiency or optimisation. In line with sociotechnical theory however, the OECD suggests that IA must consider the “non-linearity of the innovation process and its dependence upon the surrounding “system” of innovation, i.e., the institutions, actors and wider social context within which innovation happens” (ibid.). In other words, business impacts alone are not a sufficient measure for the impact of a sociotechnical system.

Furthermore, although the literature concerning impact assessment over the past forty or more years is extensive, there is no single ‘best practice’ approach to IA agreed on by all academics. Rather, there are a considerable range of IA approaches and models in circulation, each with their own merits and/or contexts. These include the widely-used life-cycle assessment model (e.g. Hauschild & Huijbregts, 2015; Kounina *et al.*, 2013), as well as other assessment models within the social (e.g. Becker, 2001; Dietz, 1987; Parent *et al.*, 2010; Vanclay, 2003), the environmental (e.g. IAIA & IEA, 1996; Jolliet *et al.*, 2003; Kates *et al.*, 1985; Monteiro & Freire, 2012; Morris & Therivel, 2001), the health (e.g. Lock, 2000; Maki *et al.*, 2008), and the economic domains (e.g. Hulme, 1997; Robinson, 1991; Rushton *et al.*, 1999). In other words, not only are there nuances to IA in terms of linearity versus sociotechnical systems, but also in terms of approaches and models that can be deployed.

In addition, in order to measure impact, organisations must identify a range of indicators to monitor the progress over time of activities and outputs ( Marr *et al*., 2004). In this regard there are also differing approaches to the identification and definition of these indicators, Parmenter (2015) suggests that there are four main types:

1. Key Result Indicators: critical results of actions indicating direction of travel and success reviewed over a longer period of time (e.g., quarterly) with no indication of how to improve results if not met
2. Key Performance Indicators: critical day-to-day management-focussed indicators reviewed over a short period of time (e.g., daily/weekly) providing indication of actions required to improve success if not met
3. Result Indicators: non-critical results of actions that underpin KRIs
4. Performance Indicators: non-critical indicators that underpin KPIs and promote alignment with organisational strategy

Parmenter (2015, p1) argues that these four types are often confused together under the title of KPI, but this can, and does, often result in “an inappropriate mix” that reduces their value and effectiveness. In the case of many large-scale EC-funded projects, where KPI monitoring is normally reported during the quarterly project plenaries, Parmenter (ibid. p.7) would argue that KRIs are a more suitable type of indicator because “a monthly, quarterly, or annual measure cannot be a KPI” and they do not necessarily suggest the actions that need to be taken if the indicator has not been met. However, this is only one view of the matter. Hence, in order to avoid becoming mired in semantic issues of definition, and because many of these indicators are a blend of existing project KPIs and impact assessment specific measures, the extended IA approach described in this paper has adopted the use of the term ‘Outcome Indicators’, based on the use of this term in the Impact Reporting and Investment Standards Core Metrics Set. In summary, IA is a complex task requiring decisions to be made by those undertaking it concerning its purpose (ex-ante – planning vs ex post – evaluation), its ontological position (linearity vs sociotechnical systems), the approach or model to use, and the exact nature of the measures to be analysed.

In addition to this broad landscape of impact assessment, there is additional extensive literature which conceptualises the IA domains for digitisation, as well as providing corresponding metrics for their measurement. One such framework as applied to the manufacturing domain identifies the following domains (with metrics in parentheses): Economy (connectivity, fostering enablers, running enterprises, improved integration); Society (smart infrastructure, digital technologies); and Industry (work digitisation, improved interaction, improved processes) ( Kotarba, 2017). However, research published around digitisation and impact assessment models for EU SMEs often focuses on one or a few aspects of digitisation and/or impact assessment, rather than understanding and discussing the relationships between them and the implications for I4.0 as a sociotechnical whole ( Isensee *et al*., 2020). Fu *et al*. (2021) further adds that there remain critical knowledge and research gaps in understanding and measuring these dimensions, and their subsequent impact on organisations themselves. This adds increasing complexity to the IA process.

In addition, turning more specifically to the manufacturing domain, the European Factories of the Future Research Association ( EFFRA, 2020) has identified a number of challenges and opportunities for EU manufacturing, especially concerning the manufacture of future products and the sustainability of manufacturing processes (in the economic, social and environmental domains), as well as the technologies and enablers which contribute to those processes. Together, these influence the digitisation and innovation research priorities. Although, enabling technologies present their own challenges and opportunities for those developing and employing them ( EFFRA, 2020; Farrand, 2014), they constitute a vital element in the manufacturing mix for emerging markets, long-term sustainability and the future competitiveness of SME manufacturers, which account for almost 40% of the manufacturing sector – a sector which employed 30.2 million EU citizens and produced €1.999 billion of valued added in total in 2019 ( Eurostat, 2020).

Pre-empting this, in 2016 the European Commission (EC) launched the “Digitizing European Industry initiative” (DEI), which was designed to reinforce the EU’s competitiveness in digital technologies within the industrial/manufacturing domain and lead to the advancement and uptake of Industry4.0 approaches across the Union. The strategy is broken down into four pillars: (1.) Digital Innovation Hubs, (2.) regulatory framework, (3.) skills, and (4.) digital platforms ( Gerrikagoitia *et al*., 2019). The EU has since launched several calls in the Horizon 2020 program to advance the development of digital industrial platforms, exceeding a €100 million of funding.

Consequently, digital platforms - as one example of enabling technologies – are an important feature of the digitalisation programme. Platforms which employ a federation approach arguably offer improved diversity and demand-led development in a wide range of industry sectors ( Silander, 2020; Silander, 2019). Examples such as Google and IBM have successfully demonstrated economic benefits by increasing net profits resulting from the creation of a quasi-federation of interrelated platforms (search, social-media, email, cloud storage, documents management, etc) ( Farrand, 2014). Furthermore, digital platforms, their methodologies, and the services/tools/products they may lead to are argued to be a key enabler for other EU challenges, for example in reducing climate change impacts and in improving education ( Silander, 2020; Silander, 2019). It is for these reasons that one pillar of the H2020 programme was devoted to digital platforms, and why impact assessment of innovation projects awarded within that programme is of interest.

Situated within this wider context, this paper presents an extended, ‘ex post’ impact assessment approach which encompasses the sociotechnical nature of the system under study based on outcome indicators as measures of impact. Such an approach can serve as a starting point for advancing IA processes in research and organizational development for advanced manufacturing. In the next section the extended approach to impact assessment within the I4.0 digital manufacturing domain will be presented.

## Method: an extended approach to impact assessment

EC H2020 I4.0 innovation projects commonly follow a generic process for establishing impact. The standard approach to measuring impact is at the beginning and end of projects within the domains of (1) industry and innovation, (2) society and the environment, and (3) the scientific community. While the objectives of projects may vary, there remain a core list of documented impact objectives that a majority of projects are expected to address in one or more ways. These include employment, value added, environmental, social, political, research and development, innovation, and education. While a majority of EU innovation projects are informed by these general H2020 domains and objectives, the IA approach presented here has extended this considerably by combining them with other best impact assessment practices, objectives, and metrics to produce an alternative methodological approach involving the alignment of impact assessment criterion with project Outcome Indicators. The IA process has been broken down into a series of Phases, ranging from scoping to mapping to data sources & collection to analysis. This phased approach enables coherence over the assessment lifetime, provides specific achievable goals, and structures process timings.

In detail, Phase 1 of this extended approach involves an online literature review of sources to identify top-level impact domains, global trends, and specific trends in whichever domain is under study, based on keyword searches and pre-existing domain knowledge. As the domain in this pilot case was I4.0 and digital manufacturing, the sources identified included the BDVA White Paper (2020) “Big Data Challenges in Smart Manufacturing,” the EFFRA Factories of the Future (2020) “Multi-annual Roadmap,” and the new Horizon Europe (2020) “Orientations towards the first strategic plan.” Next, applying emergent thematic analysis methods allows the identification of the key emergent themes most relevant to the project domain (e.g., digital manufacturing). These themes are then grouped and refined into top-level impact domains – in this case: Industrial, Technological, Social and Environmental. Thereafter, Phase 2 involves a second online literature review to identify the relevant, established impact objectives within each of the individual impact domains, resulting in a small number of the most relevant and critical objectives aligned within each of the four impact domains. Within the manufacturing domain this involved drawing from and adapting existing impact objectives from the International Association for Impact Assessment ( 2003) and the well-researched and widely applied (and recently updated) Impact Reporting & Investment Standards (IRIS) Core Metrics Set ( 2022), along with the European Commission’s Core Impact Objectives guidelines and the UN Sustainable Development Goals, although these remain relevant in non-manufacturing contexts as well. This results in a set of individual impact assessment criteria organised by top-level impact domain and linked to an impact objective. Next, Phase 3 involves the mapping of the individual impact objectives to specific, established impact metrics and to the project KPIs. This can be achieved through the application of existing metrics from the Impact Reporting and Investment Standards Catalogue of Metrics ( 2020) and from an internal literature review using existing project documentation (e.g., the Description of Action (DoA) and early project deliverable reports where project KPIs for each work package are often defined). The latter process also involves reviewing the KPIs in the project documentation to ensure they are suitable as Outcome Indicators for impact assessment and making minor adaptations where required. In a number of instances there may well be no existing KPIs in the project documentation that corresponded with the impact domain, objective, and metric. In this case new Outcome Indicators can be defined by merging the generalised approach of Kerzner (2017), the industry-specific approach detailed by Habibi *et al*. (2019), and the human-centric/effectiveness approach of Donko & Traljic (2009). At this stage the impact domains, impact objectives, impact metrics and Outcome Indicators can be listed on a spreadsheet as individual impact assessment criterion. These form the core foundation for the next phases of the Impact Assessment process.

Phase 4 then involves identifying the specific measure that would be used to assess each criterion and the data source that would be used to provide that measure. In some cases, primary data will be required, in others, data generated within the various work packages of the project will suffice. Phase 5 involves developing the data collection tools that are required for the primary data for both the baseline measure and the change measure. The baseline measurements for each impact assessment criterion and the subsequent change from that baseline constitutes change in that measurement during the lifetime of the project. The extent of the change that is measured would provide an indication of the extent of the impact of the project within that criterion. This indication can then be further enhanced with an analysis of the success in achieving (or not) the Outcome Indicator associated with that criterion. As a result, the next step is to transform the impact criterion into the most appropriate data collection format for the research context. Any primary data collection method must return results that are comparable over time and across different audiences. In this case, which meant developing and testing two quantitative, closed question surveys, with each survey question directly aligned with the impact criterion and associated Outcome Indicator (the reasons for developing two surveys will become clear in the results section). The two surveys were essentially identical in structure and questions, with certain minor tweaks to wording to be target audience appropriate. The survey design logically followed the impact domain, individual impact criterion and Outcome Indicator structure developed by this methodology, thereby ensuring that the data returned by the two baseline surveys would be consistent with the impact assessment criteria and with each other, allowing reasonable collation and analysis. The initial baseline surveys were piloted through an internal testing process with consortium members and improved and refined before release. In Phase 6 the end-of-project data collection methods should be developed, and as before they must allow ready comparison and analysis with the data collected in Phase 5. In this case, an additional two surveys were developed for the same two target audiences, with the exact same questions and structures as the baseline surveys. In addition, the participating organisations were asked that the same individual complete the baseline survey and the end-of-project survey in order to maximise data consistency over time. Once the data is returned, then the final, ex post impact assessment can take place and the results described.

**Figure 1.**
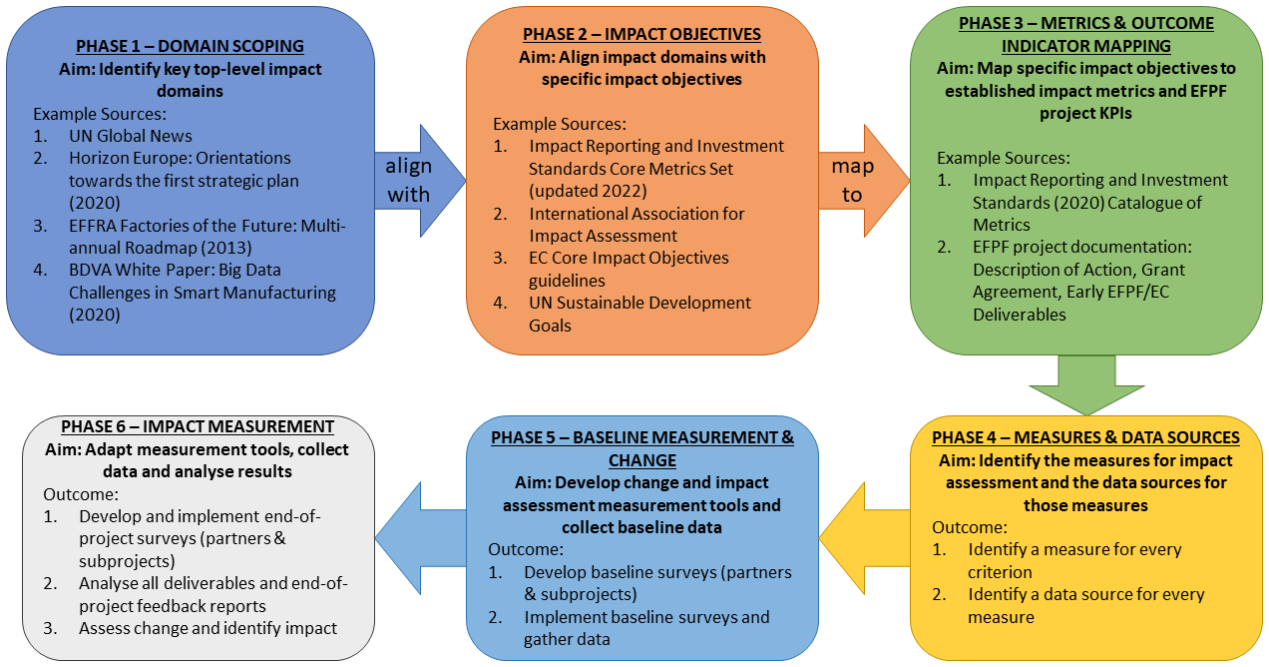
The extended impact assessment methodology.

This general, phased, extended IA approach, with specific example application in the digital manufacturing and EC H2020 context, is summarised in Figure 1 (below). Next, after a brief overview of the specific I4.0 / H2020 project in which this approach was developed and deployed, the results from this approach will be presented.

## The European Connected Factory Platform for Agile Manufacturing (EFPF) Project – an overview and acknowledgement

The extended IA approach presented in this paper was funded, developed and deployed as part of the EFPF Innovation Action project within the EC Horizon 2020 Industrial Leadership programme (#825075). EFPF is a platform which has federated five existing digital manufacturing platforms and developed new I4.0 services, thereby providing Industry 4.0 tools, services and applications via a Portal and a federated Marketplace to realise and support a connected and smart I4.0 ecosystem for SMEs and manufacturers. The platform is offered to users through a unified EFPF Portal with common interoperability standards and security protocols that allow seamless interactions and data exchange between multiple platforms, tools and services while preserving security and privacy concerns and hiding the complexity of dealing with different platform and solution providers. Interoperability has been achieved through an open data spine, designed to enable access to services, resources and solutions that were previously dispersed and therefore less time efficient. The base platform (referred to throughout as the EFPF platform) is enhanced by other additional industrial platforms, collaboration tools and smart factory systems, specifically selected to support connected factories in lot-size-one manufacturing. The EFPF platform has been made possible over four years through a €13,640,264.01 contribution from the European Commission to a consortium of 30 industrial companies, research institutions, and technology providers from ten different EU and associated states. As part of this, €2,500,000 of cascade funding was awarded to twenty industrial and SME developer subprojects to further enhance, test or validate the EFPF platform and services. These subproject users formed a very important part of the impact assessment activities. The EFPF project was launched in 2019 and will run until the end of 2022, during which time 54 EU SMEs, developers and academic institutions will have taken part both within the project consortium and through the cascade funding subprojects. It is the data collected from these organisations which is used to inform the impact assessment outcomes and to test the extended impact assessment methodology that will be detailed in the remainder of this section.

## Impact assessment results

It is important to note that the impact assessment criterion and Outcome Indicators that will be presented and analysed in this section are specifically related to impact during the lifespan of the EFPF project, as these are both measurable and within scope. It is highly anticipated that other impact effects, especially within the economic domain, will be realised post-project once greater market activity will take place. The final results of the extended approach to impact assessment were that 27 individual impact assessment criterion and associated Outcome Indicators were identified and grouped within the four impact assessment domains of Industrial (9 criteria), Technological (6 criteria), Social (8 criteria), and Environmental (4 criteria) (See Data Availability statement, Fair *et al.,* 2023). One example criterion for each impact domain can be seen in Table 1 below. It is also worth noting that the results presented here will be only a top-level overview of the impact of the EFPF project, or directly related to the individual criterion presented in Table 1. The remaining criterion and their results can be found in the EFPF Deliverable 10.11 once it is made available by the EC.

**Table 1.** Example European Connected Factory Platform for Agile Manufacturing (EFPF) impact assessment criteria after the implementation of the extended impact assessment methodology.

Domain	Impact Objective	IRIS metric	Measure / Survey Question	Data Source	Outcome Indicators
Industrial	Industrial Leadership	New Firms Entered (OI3141)	Number of new SMEs/ businesses with products available on the EFPF Marketplace and/or federated marketplaces	Count of new businesses with products available on the EFPF (and/or federated) marketplace	>12 new businesses with products on the EFPF/ federated marketplaces and/or services available on the EFPF Portal
Technology	Uptake of New Technologies	Client Organisations: Provided New Access (PI2575)	Since joining the EFPF project, has your organisation made use of any new EFPF services/ products that were previously unavailable to you?	Baseline surveys / End-of-project surveys / subproject deliverable data	>75% of respondents report that the EFPF platform has provided them with new access to products/services previously unavailable to them
Social	Education Systems & Upskilling	Individuals Trained: Technical Assistance (PI5352)	How many people have received EFPF training or technical assistance on relevant development topics (e.g. NOT open call application support webinars….etc)	Baseline surveys / End-of-project surveys / Number of issues raised on Tiki	>75% of respondents report receiving training/ technical assistance by the end of the project >30 Tiki issues raised by subprojects
Environmental	Climate Change Mitigation	Greenhouse Gas Emissions Avoided (PI2764)	Number of EFPF plenaries and project meetings/events attended virtually	Count of number of virtual events and virtual attendees	6 plenaries and events attended virtually by all attendees

Two different surveys were developed to be suitable for distribution to, in the first case, EFPF consortium partners, and in the second case, to the cascade funding subprojects, although both surveys differed only in some minor linguistic tweaks. A total of 32 SMEs from nine different EU countries participated in the baseline survey represented by individuals from their management structures. Participating organisations represented domains including manufacturing and engineering, industrial processes, electronics and systems, information and communication, software development and others, and ranged in size from micro enterprises (fewer than 10 employees) to large businesses (over 250 employees), with annual turnovers ranging from €300,000 to €5million. The same respondents plus an additional 14 first-time respondents, with the same or similar organisational profiles, completed the end-of-project surveys, giving a total number of respondents across the impact assessment of 78. The surveys were created and hosted online via the University of Southampton iSurvey tool. The baseline survey was deployed as part of Phase 5 from October-November 2021 and the end-of-project surveys in Phase 6 were deployed from September to November 2022.

### Industrial domain

The key impact objectives for the Industrial Domain were identified during Phase 2 as: Industrial Leadership, Data Integration and Network Building. Analysis of the results indicated that the EFPF project has significantly impacted the Industrial Domain by contributing in concrete, measurable ways to the three impact objectives through:

- providing business innovations in tools, services, applications, and I4.0 standards
- increasing value to I4.0 stakeholders
- enabling new market entrants and new I4.0 solution users
- expanding marketplace linkages and integration
- providing new access to integration and business opportunities for I4.0 stakeholders.

Specifically, in relation to the criterion in Table 1 above the results were as follows ( Table 2):

**Table 2.** Results for a single industrial domain impact assessment criterion.

Domain	Impact Objective	IRIS metric	Measure / Survey Question	Data Source	Outcome Indicators	Outcome Indicator Results	Baseline	% change from baseline
Industrial	Industrial Leadership	New Firms Entered (OI3141)	Number of new SMEs/businesses with products available on the EFPF Marketplace and/or federated marketplaces	Count of new businesses with products available on the EFPF (and/or federated) marketplace	>12 new businesses with products on the EFPF/federated marketplaces and/or services available on the EFPF Portal	15 new businesses with products / services on EFPF Marketplace and Portal	0	100%

This criterion demonstrates a 100% increase (a significant impact) from the baseline measure as a result of EFPF activity enabling 15 new SME/businesses to market their tools, services, or applications via the federated EFPF marketplace, thereby meeting the impact metric of New Firms Entered, which indicates Industrial Leadership within the Industrial domain.

### Technological dmain

The key impact objectives for the Technological Domain were identified during Phase 2 as: Uptake of New Technologies, Open Science, Data Integration and Network Building. Analysis of the results indicated that the EFPF project has significantly impacted the Technological Domain by contributing in concrete, measurable ways to the four impact objectives through:

- providing access to new I4.0 tools, services, and applications
- expanding the SME (and other) client base
- providing and publishing open access and open source I4.0 software
- providing business innovation through integrated and validated tools, services, and applications
- fostering knowledge and technology exchange with other I4.0 initiatives.

Specifically, in relation to the criterion in Table 1 above the results were as follows ( Table 3):

**Table 3.** Results for a single technological domain impact assessment criterion.

Domain	Impact Objective	IRIS metric	Measure / Survey Question	Data Source	Outcome Indicators	Outcome Indicator Results	Baseline	% change from baseline
Technology	Uptake of New Technologies	Client Organisations: Provided New Access (PI2575)	Since joining the EFPF project, has your organisation made use of any new EFPF services/ products that were previously unavailable to you?	Baseline surveys / End-of- project surveys / subproject deliverable data	>75% of respondents report that the EFPF platform has provided them with new access to products/ services previously unavailable to them	93% report access to new products / services	43%	+50%

This criterion demonstrates a 50% increase (a significant impact) from the baseline measure as a result of EFPF activity enabling new access to I4.0 tools, services, or applications, thereby meeting the impact metric of Client Organisation Provided New Access, which indicates the Uptake of New Technologies within the Technological domain.

### Social Domain

The key impact objectives for the Social Domain were identified during Phase 2 as: Knowledge Sharing, Education Systems & Training, and Open Science. Analysis of the results indicated that the EFPF project has significantly impacted the Social Domain by contributing in concrete, measurable ways to the three impact objectives through:

- engaging a large number of stakeholders in a wide range of ways over a sustained period
- providing effective training and technical assistance to large numbers of groups and individuals over a sustained period
- publishing academic papers in open access journals.

Specifically, in relation to the criterion in Table 1 above the results were as follows ( Table 4):

**Table 4.** Results for a single Social Domain impact assessment criterion.

Domain	Impact Objective	IRIS metric	Measure / Survey Question	Data Source	Outcome Indicators	Outcome Indicator Results	Baseline	% change from baseline
Social	Education Systems & Upskilling	Individuals Trained: Technical Assistance (PI5352)	How many people have received EFPF training or technical assistance on relevant development topics (e.g. NOT open call application support webinars….etc)	Baseline surveys / End-of- project surveys / Number of issues raised on Tiki	>75% of respondents report receiving training/technical assistance by the end of the project >30 Tiki issues raised by subprojects	81% report receiving training / assistance 140 Tiki issues raised	67%	+14%

This criterion demonstrates a 14% increase (a minor impact) from the baseline measure as a result of EFPF activity enabling training or technical assistance to stakeholders, thereby meeting the impact metric of Individual Trained: Technical Assistance, which indicates the Upskilling within the Social domain.

### Environmental Domain

The key impact objectives for the Environmental Domain were identified during Phase 2 as: Energy Efficiency, Waste Reduction and Climate Change Mitigation. Analysis of the results indicated that the EFPF project has impacted the Environmental Domain by contributing in concrete, measurable ways to the three impact objectives through:

- enabling energy consumption reductions
- enabling waste production reductions
- enabling greater product lifecycle circularity
- saving greenhouse gas emissions.

Specifically, in relation to the criterion in Table 1 above the results were as follows ( Table 5):

**Table 5.** Results for a single Environmental Domain impact assessment criterion.

Domain	Impact Objective	IRIS metric	Measure / Survey Question	Data Source	Outcome Indicators	Outcome Indicator Results	Baseline	% change from baseline
Environmental	Climate Change Mitigation	Greenhouse Gas Emissions Avoided (PI2764)	Number of EFPF plenaries and project meetings/ events attended virtually	Count of number of virtual events and virtual attendees	6 plenaries and events attended virtually by all attendees	9 large project meetings held virtually for all attendees	0	+100%

This criterion demonstrates a 100% increase (a significant impact) from the baseline measure as a result of EFPF holding five plenaries, two large technical meetings and two EC review meetings virtually, thereby meeting the impact metric of Greenhouse Gas Emissions Avoided (through over 200 fewer short-haul flights), which indicates Climate Change Mitigation within the Environmental domain.

## Conclusion

This paper has introduced a phased, extended, ex post, sociotechnical approach to Impact Assessment within the I4.0 and digital manufacturing domain situated in an EC-funded H2020 context. This approach was developed and deployed in the EFPF H2020 project. It has successfully enabled the alignment and mapping of impact assessment domains, objectives, and metrics with EFPF project KPIs and impact assessment Outcome Indicators and identified measures and data sources for each criterion, and in so doing has provided a more extensive impact assessment than is typically expected within an EC H2020 project. The methodology detailed in this paper has proven effective in returning concrete, measurable results across 27 individual impact assessment criteria (see previous section). Furthermore, the extended impact assessment methodology is also effective in enabling a compelling narrative to be structured around the impact story for the purposes of reporting and/or dissemination.

It is hoped that this extended approach may also be replicable by other large-scale EU-funded projects and/or other organisations within the manufacturing domain, as it is not project-specific nor bounded by a single set of outcome indicators or individual criterion. Finally, the end results of the impact assessment process will further contribute to a growing number of studies that indicate the extent to which enabling technologies – such as the EFPF platform – add value or result in tangible benefits to EU SME manufacturers. It is further hoped that this extended approach to impact assessment will prove useful given the EC’s increasing emphasis on Industry5.0 concepts of resilience, sustainability and human-centric technologies and the notion of prosperity – in other words manufacturing as a sociotechnical system. It will be increasingly important that organisations, whether part of an innovation project or not, are able to effectively assess their impact as part of this wider sociotechnical system. By adapting the approach described here to other organisational contexts and demands, organisations and projects will be able to reliably monitor and evaluate their actions in relation to I5.0 drivers and demonstrate to customers and authorities the impact their operations have on the wider community, society, and environment. In this way, open impact assessment becomes a source of value to an organisation as it can foster increased trustworthiness between them, their customers, their supply chains, and the wider public.

## Ethics

This research consisted of data anonymously collected from project partners (consortium members and Open Call subprojects) under University of Southampton ERGO ethics approval number 87358.

## Data Availability

University of Southampton PURE: An extended approach to impact assessment in the Horizon 2020 digital manufacturing domain. https://doi.org/10.5258/SOTON/D2629 ( Fair *et al.,* 2023) The project contains the following underlying data: EFPF_Impact_Assessment_Data-RAW Data are available under the terms of the Creative Commons Attribution 4.0 International license (CC BY 4.0).
