# Supplementary material for: An extended approach to impact assessment in the Horizon 2020 digital manufacturing domain
Source: Open Res Eur. 2024 Jan 8;4:9. [Version 1] doi: 10.12688/openreseurope.16235.1 (PMC11128048; doi:10.12688/openreseurope.16235.1)
Supplement: Peer review history

# Reviewer report: Reviewer response for version 1

**DOI:** 10.21956/openreseurope.17528.r37888

**Published:** 2024-05-25

**Authors:** Davide Settembre Blundo

**Affiliations:** 1 Rey Juan Carlos University, Madrid, Spain

This paper presents a valuable contribution to the field of impact assessment in large-scale, EU-funded manufacturing projects. I appreciate the authors' efforts to develop a six-phase extended approach that goes beyond traditional methods, offering a more detailed and comprehensive assessment process. The alignment of project outcome indicators with impact assessment criteria through a review and integration of existing frameworks is a commendable approach, and the application in the EFPF project demonstrates its potential utility.

 However, I have a few suggestions for improvement before publication:

**1. Typos:** While I haven't identified specific typos, a thorough proofread is recommended to ensure the final manuscript is free of any errors.

**2. Generalizable Protocol:** While the authors describe the six-phase process, it would be highly beneficial to further develop this into a generalizable protocol. This would facilitate the replication and application of the extended approach to other contexts beyond the specific EFPF project. This could involve providing detailed steps, templates, and tools for each phase, making the approach more accessible and user-friendly for other researchers and practitioners.

**3. Specificity of Impact:** While the authors report that 24 of 27 IA criteria were met or exceeded, further details on the specific impacts achieved would be valuable. This could include concrete examples, data visualizations, or qualitative assessments from stakeholders. Providing more specific evidence of the project's contribution to each priority area would strengthen the overall impact narrative.

 Overall, this paper presents a promising approach with potential for wider application. By addressing the suggested improvements, the authors can further enhance the clarity, replicability, and impact of their work.

 I recommend a short update to address the above points. Once these have been addressed, this paper is ready for publication and can be a valuable resource for researchers and practitioners working on impact assessment in large-scale production projects.

Is the rationale for developing the new method (or application) clearly explained?

Yes

Is the description of the method technically sound?

Yes

Are the conclusions about the method and its performance adequately supported by the findings presented in the article?

Yes

If any results are presented, are all the source data underlying the results available to ensure full reproducibility?

Yes

Are sufficient details provided to allow replication of the method development and its use by others?

Partly

Reviewer Expertise:

Innovation & Sustainability Management, Manufacturing, Commodity Sciences

I confirm that I have read this submission and believe that I have an appropriate level of expertise to confirm that it is of an acceptable scientific standard.

# Reviewer report: Reviewer response for version 1

**DOI:** 10.21956/openreseurope.17528.r39370

**Published:** 2024-05-06

**Authors:** Devarajan Ramanujan

**Affiliations:** 1 Aarhus University, Aarhus, Denmark

This article presents an approach for conducting impact assessments in large European Union funded projects. The proposed approach is applied to the European Connected Factory Platform for Agile Manufacturing project funded by the European Commission. The article highlights a significant existing limitation common to several large innovation research projects, i.e., the lack of a systematic approach for framing and monitoring the resulting impacts. This gap is especially relevant for technology-oriented projects as it can be challenging to assess their societal and environmental impacts.

 The article presents a good overview of prior literature in this domain and motivates the need for a more thorough impact assessment approach in the digital manufacturing domain. The review of the different scopes, assessment methods, and performance/results indicators in the context of impact assessment is useful for readers in the digital manufacturing domain. While the presented methodology has merit, a more detailed description of the methodology will increase its adoption by the research community. Dividing the methodology into the presented six phases supports a more systematic approach towards impact assessment. However, the procedure within each phase is detailed in a generic manner. Although exemplary references are provided for certain phases, they may not be applicable to other projects, and the selection of the objectives and indicators is left to the user, with limited guidance on how to do so.

 The case study demonstrates that the proposed methodology can be applied in the real world. However, little detail is provided on the rationale for selecting the specific objectives and indicators. Furthermore, the outcome indicators appear to focus on “internal performance” without sufficient external validation. For example, it can be argued that apart from the number of businesses signing up for the platform, the actual impact of the platform should be validated e.g., in terms of the increased profitability, sustainability, competitiveness. Similarly, the importance of measuring climate change mitigation due to avoided project meetings cannot be justified without validating other impacts due to the project (e.g., increased use of computing resources, increased resource use due to higher sales). Therefore, a more nuanced reflection and refinement the selected impact measurement criterion is warranted (e.g., after Phase 6 in the methodology).

 Finally, the article would benefit from through proof-reading. There are several instances where abbreviations are not expanded during their first use. Please also refrain from unnecessary capitalization.

Is the rationale for developing the new method (or application) clearly explained?

Yes

Is the description of the method technically sound?

Yes

Are the conclusions about the method and its performance adequately supported by the findings presented in the article?

Yes

If any results are presented, are all the source data underlying the results available to ensure full reproducibility?

Partly

Are sufficient details provided to allow replication of the method development and its use by others?

Partly

Reviewer Expertise:

Sustainable manufacturing, Manufacturing digitalisation, Engineering Design

I confirm that I have read this submission and believe that I have an appropriate level of expertise to confirm that it is of an acceptable scientific standard, however I have significant reservations, as outlined above.

# Reviewer report: Reviewer response for version 1

**DOI:** 10.21956/openreseurope.17528.r38674

**Published:** 2024-05-02

**Authors:** Fotios K. Konstantinidis

**Affiliations:** 1 Department of Production and Management Engineering, Institute of Communication and Computer Systems, Athens, Greece

Industry 5.0 primarily emphasizes sustainability, agility, and human-centricity, as outlined in reports provided by the EC. In the introduction section, it would be beneficial to align the provided text within these pillars.

 At the conclusion of the related work, I propose incorporating bullet points summarizing the main contributions of this paper, serving to introduce the subsequent content to the reader.

 Given that this paper encompasses a broader scope beyond manufacturing, I suggest renaming the section from "The European Connected Factory Platform for Agile Manufacturing (EFPF) Project – an overview and acknowledgment" to "Case Study: The European Connected Factory Platform for Agile Manufacturing (EFPF) Project". Additionally, quantitative characteristics of the SMEs involved in this analysis should be included (moving the relevant text from the subsequent paragraph "Two different... ... November 2022"). However, the focus will not be on financial numbers related to the EFPF platform.

 There is a spelling error in the phrase "Technological domain."

 Before the conclusion, I recommend adding a discussion section where the results of previous domains will be analyzed, providing insights.

Is the rationale for developing the new method (or application) clearly explained?

Yes

Is the description of the method technically sound?

Partly

Are the conclusions about the method and its performance adequately supported by the findings presented in the article?

Yes

If any results are presented, are all the source data underlying the results available to ensure full reproducibility?

Partly

Are sufficient details provided to allow replication of the method development and its use by others?

Yes

Reviewer Expertise:

Smart Manufacturing PhD, Expert on Indusry 4.0

I confirm that I have read this submission and believe that I have an appropriate level of expertise to confirm that it is of an acceptable scientific standard, however I have significant reservations, as outlined above.
